# Empowering Partnerships Among Researchers, Persons Living With Dementia, and Caregivers

**DOI:** 10.1093/geroni/igaa057.2587

**Published:** 2020-12-16

**Authors:** Heidi Gil, Stephani Shivers, Bob Savage, Geri Taylor, Jim Taylor, Erica DeFrancesco, Maria O’Connell, Michael Lepore

**Affiliations:** 1 LiveWell Alliance, LiveWell Alliance, Connecticut, United States; 2 LiveWell Alliance, Plantsville, Connecticut, United States; 3 Dementia Peer Coalition, Plantsville, Connecticut, United States; 4 The Taylors, New York City, New York, United States; 5 LiveWell Dementia Specialists, Plantsville, Connecticut, United States; 6 Yale School of Medicine, New Haven, Connecticut, United States; 7 LiveWell Alliance, Southington, Connecticut, United States

## Abstract

Conducting research collaboratively with persons living with dementia (PLWD) requires preparation. To enhance the capacity of PLWD to be engaged in research, we collaboratively developed and deployed a replicable two-day training workshop for PLWD, care partners, and professional/academic researchers. An assessment of strengths, values and experiences, and an introductory education session, served as experiential warmup activities for PLWD prior to the two-day workshop. Twelve PLWD, nine care partners, and 14 professional/academic researchers participated in the workshop. Participants identified 16 topics as highly important for research and prioritized four topics for immediate study design. Studies addressing these topics were collaboratively designed by PLWD, care partners, and professional researchers during day two of the workshop. Lessons learned from the workshop, including key preparation steps for collaboration—such as securing minimally distracting collaboration environments—are summarized, and access to a free toolkit that supports workshop replication is provided.

